# A Prehospital Triage System to Detect Traumatic Intracranial Hemorrhage Using Machine Learning Algorithms

**DOI:** 10.1001/jamanetworkopen.2022.16393

**Published:** 2022-06-10

**Authors:** Daisu Abe, Motoki Inaji, Takeshi Hase, Shota Takahashi, Ryosuke Sakai, Fuga Ayabe, Yoji Tanaka, Yasuhiro Otomo, Taketoshi Maehara

**Affiliations:** 1 Department of Neurosurgery, Tokyo Medical and Dental University, Tokyo, Japan; 2 Institute of Education, Tokyo Medical and Dental University, Tokyo, Japan; 3 Department of Acute Critical Care and Disaster Medicine, Graduate School of Medical and Dental Sciences, Tokyo Medical and Dental University, Tokyo, Japan

## Abstract

**Importance:**

An adequate system for triaging patients with head trauma in prehospital settings and choosing optimal medical institutions is essential for improving the prognosis of these patients. To our knowledge, there has been no established way to stratify these patients based on their head trauma severity that can be used by ambulance crews at an injury site.

**Objectives:**

To develop a prehospital triage system to stratify patients with head trauma according to trauma severity by using several machine learning techniques and to evaluate the predictive accuracy of these techniques.

**Design, Setting, and Participants:**

This single-center retrospective cohort study was conducted by reviewing the electronic medical records of consecutive patients who were transported to Tokyo Medical and Dental University Hospital in Japan from April 1, 2018, to March 31, 2021. Patients younger than 16 years with cardiopulmonary arrest on arrival or with a significant amount of missing data were excluded.

**Main Outcomes and Measures:**

Machine learning–based prediction models to detect the presence of traumatic intracranial hemorrhage were constructed. The predictive accuracy of the models was evaluated with the area under the receiver operating curve (ROC-AUC), area under the precision recall curve (PR-AUC), sensitivity, specificity, and other representative statistics.

**Results:**

A total of 2123 patients (1527 male patients [71.9%]; mean [SD] age, 57.6 [19.8] years) with head trauma were enrolled in this study. Traumatic intracranial hemorrhage was detected in 258 patients (12.2%). Among several machine learning algorithms, extreme gradient boosting (XGBoost) achieved the mean (SD) highest ROC-AUC (0.78 [0.02]) and PR-AUC (0.46 [0.01]) in cross-validation studies. In the testing set, the ROC-AUC was 0.80, the sensitivity was 74.0% (95% CI, 59.7%-85.4%), and the specificity was 74.9% (95% CI, 70.2%-79.3%). The prediction model using the National Institute for Health and Care Excellence (NICE) guidelines, which was calculated after consultation with physicians, had a sensitivity of 72.0% (95% CI, 57.5%-83.8%) and a specificity of 73.3% (95% CI, 68.7%-77.7%). The McNemar test revealed no statistically significant differences between the XGBoost algorithm and the NICE guidelines for sensitivity or specificity (*P* = .80 and *P* = .55, respectively).

**Conclusions and Relevance:**

In this cohort study, the prediction model achieved a comparatively accurate performance in detecting traumatic intracranial hemorrhage using only the simple pretransportation information from the patient. Further validation with a prospective multicenter data set is needed.

## Key Points

**Question:**

Can machine learning algorithms be used to triage patients with head trauma according to their severity before transportation?

**Findings:**

In this cohort study of 2123 patients with head trauma, a machine learning–based prediction model detected traumatic intracranial hemorrhage with a sensitivity of 74% and a specificity of 75% by using only the patient’s prehospital information.

**Meaning:**

This study suggests that a machine learning algorithm can accurately stratify patients with head trauma according to severity in prehospital settings and may improve the prognosis of patients with severe traumatic head injury.

## Teaser

This cohort study describes the development of a prehospital triage system to stratify patients with head trauma according to trauma severity by using several machine learning techniques and evaluates the predictive accuracy of these techniques.

## Introduction

Traumatic brain injury (TBI) is common in neurosurgery practice. Approximately 10 million to 69 million people worldwide experience TBI annually, and the number of patients with TBI is increasing.^1,2,3^ Although approximately 70% to 90% of all TBI cases are mild and necessitate neither admission nor neurosurgical intervention,^4^ moderate or severe TBI can become a major cause of death or severe disability. Because early efficient treatments during the acute period of TBI improve patient outcomes,^5^ it is important to adequately stratify patients with head trauma according to their trauma severity.

To this end, several screening methods to identify patients with clinically important brain injuries have been proposed,^6,7,8^ and numerous reports have supported the reliability of these screening tools.^9,10,11^ However, because these screening tools were developed for physicians’ use, they cannot be used for field triage. The prehospital triage of patients with TBI is important because it eliminates any delays in care, decreases the need for patient transportation between hospitals, and allows for streamlining of the medical system. However, to our knowledge, there have been no established protocols for stratifying patients with TBI at the scene before transportation.

In recent years, research on clinical event prediction using machine learning algorithms has been actively conducted, and more complicated and reliable classification methods have become possible.^12,13,14,15,16,17,18,19,20^ Liu et al^21^ developed a machine learning triage system that can be used to detect severity of patients’ injuries using basic clinical information. Therefore, we hypothesized that machine learning approaches might make it possible to produce reliable prediction models even when using insufficient information that was collected on the scene.

The purpose of this study was to create a prehospital triage system that can discriminate patients with traumatic intracranial hemorrhage (tICH) from those without tICH. By using several representative machine learning algorithms, we built prediction models of tICH for patients with head trauma. We verified the models’ prediction performance by applying these models on test data, and we compared these models with the conventional screening tools.

## Methods

### Study Population

**Figure 1.**
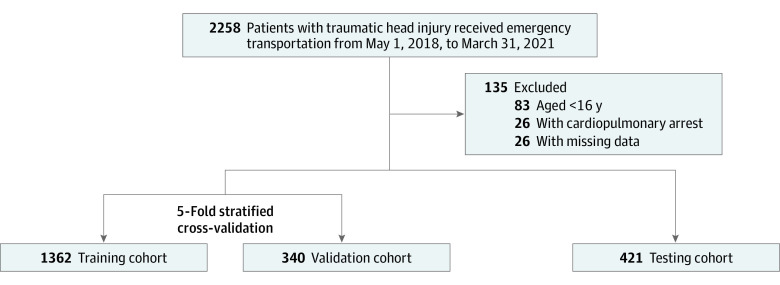
Study Flowchart

We retrospectively reviewed the electronic medical records of patients who were transported to Tokyo Medical and Dental University Hospital in Japan for head trauma from April 1, 2018, to March 31, 2021. Based on this retrospective review, we enrolled 2258 patients with head trauma who underwent head computed tomography (CT). Patients younger than 16 years (n = 83) with cardiopulmonary arrest at the time of emergency service contact (n = 26) or with missing data in more than 2 of the features (n = 26) were excluded, leaving 2123 patients included in this analysis (Figure 1). This study was approved by the institutional review board of Tokyo Medical and Dental University, which approved a waiver of patient consent because this is a retrospective study and all data were deidentified. This study followed the Transparent Reporting of a Multivariable Prediction Model for Individual Prognosis or Diagnosis (TRIPOD) reporting guideline.

### Outcome

The predicted outcome was the presence of tICH on results of initial head CT scanning, where tICH was defined as traumatic subarachnoid hemorrhage, acute subdural hematoma, acute epidural hematoma, or cerebral contusion at the time of consultation. The radiologic findings were double-checked by either a radiologist and a neurosurgeon or a radiologist and an emergency medicine physician.

### Features for Prediction Model

We obtained data from the electronic medical records of patients at the Tokyo Medical and Dental University Hospital and selected the following 18 features as explanatory variables, which are routinely collected by ambulance crews in Japan: the patient’s age, sex, systolic blood pressure, heart rate, body temperature, respiratory rate, state of consciousness (assessed with the eye component of the Glasgow Coma Scale^22^ [E-GCS; eAppendix in the Supplement] and disorientation), pupil abnormalities (defined as anisocoria or bilateral pupil dilation and loss of light reflex), high-energy head trauma (head trauma by dangerous mechanisms as defined in the National Institute for Health and Care Excellence [NICE] guidelines; eAppendix in the Supplement),^23^ head trauma scars, multiple trauma (combination of head trauma and other traumas to the body), posttraumatic seizures, loss of consciousness, vomiting, hemiplegia, and clinical deterioration, known as “talk and deteriorate,”^24,25,26^ which is defined in this study as a reduction in the E-GCS score of 1 point or more before transportation from the site.

### Data Preprocessing

The missing data for the body temperature and respiratory rate were filled with the overall mean value of each category. Patients with missing data other than body temperature and respiratory rate were excluded. The values of each feature were normalized for the support vector machine and logistic regression.

### Machine Learning Algorithms

To build the classification models, we used 5 representative machine learning algorithms (eg, extreme gradient boosting [XGBoost], random forest, support vector machine [linear kernel and radial basis function kernel] and logistic regression) as prediction model–based algorithms.

The XGBoost algorithm is an efficient implementation of gradient tree boosting, which is a representative ensemble learning algorithm that is based on decision trees.^27,28^ In the algorithm, weak learners are trained successively to split the residual of the last prediction. In addition, adding a regularization term to the loss function results in creating a strong learner without overfitting.

Random forest^29,30^ is also an ensemble classifier that is based on decision trees. In the algorithm, a large number of weak learners are trained using randomly bootstrapped data. Each weak learner votes for its predicted outcome, and the most voted class is adopted as the prediction result of all learners.

Support vector machine is a supervised machine learning algorithm in which a binary classification boundary is set to maximize the margin from each data sample. Logistic regression^31^ is one of the most widely used classification algorithms. It is used to compute the probability of an occurrence of binary outcomes using the sigmoid function.

To build the classification models based on these 5 machine learning algorithms, we used Python, version 3.6 (Python Software Foundation) and several Python modules (numpy, sklearn, matplotlib, pandas, and xgboost). In brief, we used the values shown in the eTable in the Supplement for each hyperparameter of the machine learning algorithms. For the other parameters, we used the default values. To validate the classification models and to optimize the parameters in the models, we performed a 5-fold cross-validation.

### Training, Cross-Validation, and Test

We used 80% of the data to train the classification models through a 5-fold stratified cross-validation. The remaining 20% of the data were reserved to test the predictive ability of the resultant classification models. In the process of cross-validation, scikit-learn, version 0.23.2 (scikit-learn Developers) was used to implement a grid search to obtain the optimal hyperparameters. The hyperparameters were tuned to maximize the area under the receiver operator characteristic curve (ROC-AUC). The trained classifier models calculated 2 class probabilities (the probability for the class with tICH and the probability for the class without tICH) for each patient. We used the Platt calibration^32,33^ to calibrate the class probabilities from each classifier model.

As shown in Table 1, our data set was imbalanced owing to the relative rarity of tICH among patients with head injury. Therefore, to solve the issues associated with the imbalanced data set, we introduced class weights to modify the loss function; that is, in a similar manner to Dong et al,^34^ we assigned a 10-fold higher weight to the positive cases (cases with tICH) than the negative cases (cases without tICH).

**Table 1.** Baseline Features of the Study Cohort

Feature	Patients, No. (%)		*P* value
	Training and validation cohort (n = 1702)	Testing cohort (n = 421)	
Age, mean (SD), y	57.5 (19.8)	58 (19.9)	>.99
Sex			
Male	1220 (71.7)	307 (72.9)	>.99
Female	482 (28.3)	114 (27.1)	
Score on the eye component of the GCS			
4	1558 (91.5)	387 (91.9)	>.99
3	82 (4.8)	13 (3.1)	
2	12 (0.7)	3 (0.7)	
1	50 (2.9)	18 (4.3)	
Disorientation			
Yes	594 (34.9)	139 (33.0)	>.99
No	1108 (65.1)	282 (67.0)	
Vital signs, mean (SD)			
Systolic blood pressure, mm Hg	130.4 (27.6)	129.2 (26)	>.99
Heart rate, beats/min	88.2 (17.5)	88.3 (17.7)	>.99
Body temperature, °C	36.6 (8.0)	36.4 (1.6)	>.99
Respiratory rate, breaths/min	18.7 (4.2)	18.5 (2.4)	>.99
Pupil abnormality			
Yes	13 (0.8)	5 (1.2)	>.99
No	1689 (99.2)	416 (98.8)	
Hemiparesis			
Yes	12 (0.7)	6 (1.4)	>.99
No	1690 (99.3)	415 (98.6)	
Posttraumatic seizure			
Yes	14 (0.8)	7 (1.7)	>.99
No	1688 (99.2)	414 (98.3)	
Vomiting			
Yes	65 (3.8)	9 (2.1)	>.99
No	1637 (94.2)	412 (97.9)	
Loss of consciousness			
Yes	63 (3.7)	13 (3.1)	>.99
No	1639 (96.3)	408 (96.9)	
Talk and deteriorate			
Yes	8 (0.5)	3 (0.7)	>.99
No	1694 (99.5)	418 (99.3)	
High-energy head trauma			
Yes	181 (10.6)	36 (8.6)	>.99
No	1521 (89.4)	385 (91.4)	
Head trauma scar			
Yes	1366 (80.3)	334 (79.3)	>.99
No	336 (19.7)	87 (20.7)	
Multiple trauma			
Yes	707 (41.5)	169 (40.1)	>.99
No	995 (58.5)	252 (59.9)	
Alcohol intake			
Yes	841 (49.4)	200 (47.6)	>.99
No	861 (50.6)	221 (52.4)	
Outcome: tICH			
Yes	208 (12.2)	50 (11.9)	>.99
No	1494 (87.8)	371 (88.1)	

Abbreviations: GCS, Glasgow Coma Scale; tICH, traumatic intracranial hemorrhage.

### Performance Evaluation

To evaluate the predictive performance of the classifier models, we calculated 6 representative performance evaluation measures, including sensitivity, specificity, positive predictive value (PPV), negative predictive value, positive likelihood ratio, and negative likelihood ratio. The ROC-AUC and the area under the precision recall curve (PR-AUC) were also computed. The recall corresponds to the sensitivity, and the precision corresponds to the PPV. The PR curve is often used along with the ROC curve to assess the model performance, especially in imbalanced data sets.

### Statistical Analysis

The statistical analysis was conducted using R, version 4.0.3 (R Group for Statistical Computing). Mann-Whitney tests were used to analyze nonnormally distributed variables, and the χ^2^ test was chosen for the analysis of categorical data. To compare the sensitivity and specificity between each model, the McNemar test was used. All *P* values were from 2-sided tests and results were deemed statistically significant at *P* < .05. Bonferroni adjustment was applied for multiple comparison testing.

## Results

### Baseline Characteristics

This study cohort included 2123 patients (1527 male patients [71.9%]; mean [SD] age, 57.6 [19.8] years; age range, 16-102 years), of whom 258 (12.2%) were found to have tICH. All of the patients were divided into a training set (1702 cases, 80.2% of all data) and a testing set (421 cases, 19.8% of all data) by a random allocation that was stratified by tICH. Table 1 shows the breakdown of each explanatory feature in each group. No statistically significant difference was found between the 2 groups in any of the features.

### Comparison Among the Machine Learning Algorithms in the Cross-Validation

**Figure 2.**
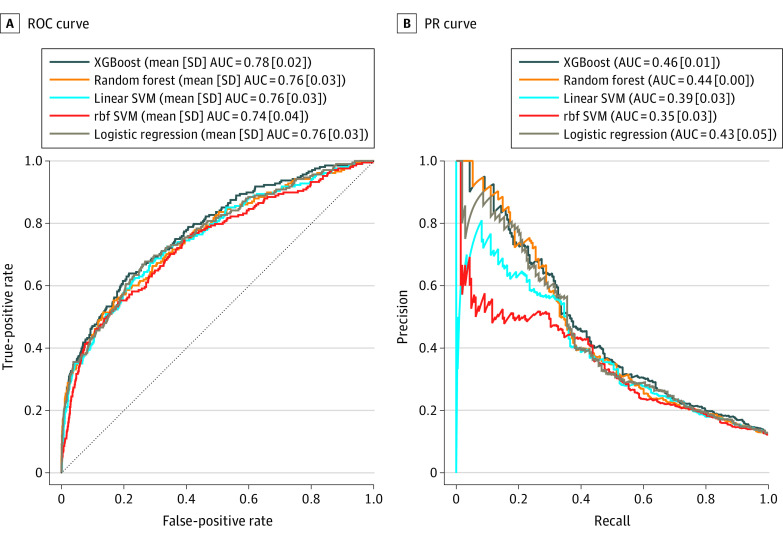
Receiver Operating Characteristic (ROC) Curves and Precision Recall (PR) Curves for Each Machine Learning Algorithm in the 5-Fold Stratified Cross-Validation The diagonal dotted line represents the random classifier. AUC indicates area under the curve; rbf, radial basis function; SVM, support vector machine; and XGBoost, extreme gradient boosting.

Figure 2 shows the ROC and PR curves for the 5 machine learning algorithms. Among the algorithms, XGBoost achieved the highest mean (SD) AUC in both the ROC curve (0.78 [0.02]) and PR curve (0.46 [0.01]). Therefore, we selected XGBoost as an appropriate algorithm to develop the prediction models for the patient stratification and conducted a further analysis with it for its predictive validity.

### Prediction Model for the Test Data Using XGBoost

**Figure 3.**
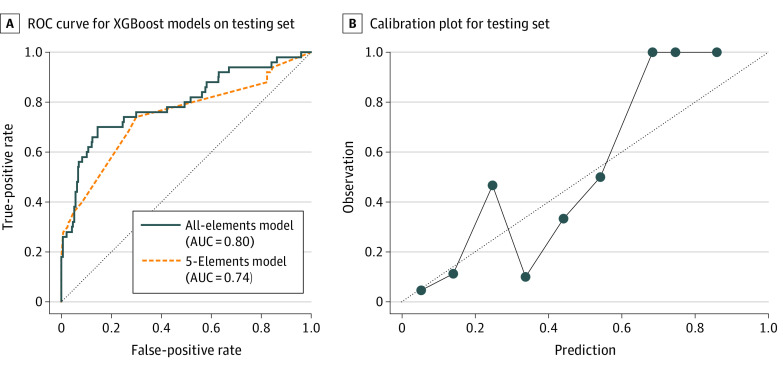
Receiver Operating Characteristic (ROC) Curve for the Extreme Gradient Boosting (XGBoost) Models on the Testing Set and Calibration Plot for the Testing Set Diagonal dotted lines represent the random classifier (A) and perfectly calibrated plot (B). AUC indicates area under the curve.

To classify the patients with tICH, we built a classifier model based on the XGBoost algorithm with all 18 features and named this model the “all-elements model.” This model achieved a mean (SD) ROC-AUC of 0.78 (0.02) and a mean (SD) PR-AUC of 0.46 (0.01) for cross-validation (eFigure 1 in the Supplement) and an ROC-AUC of 0.80 and a PR-AUC of 0.51 for the testing set (Figure 3; eFigure 2 in the Supplement). A calibration plot for the testing set is shown in Figure 3.

To quantify the association of the 18 features with the outcomes, we performed an analysis of the importance of the features for the all-elements model. As shown in eFigure 3 in the Supplement, among the 18 features, 5 variables (disorientation, high-energy head trauma, head trauma scar, E-GCS, and pupil abnormality) were likely to be associated with obtaining accurate predictions.

Based on the results of this feature importance analysis and to simplify the classification models, we selected the 5 most important features (disorientation, high-energy head trauma, head trauma scar, E-GCS, and pupil abnormality) and built another classification model (named the “5-elements model”) with the selected features. The 5-elements model showed a mean (SD) ROC-AUC of 0.74 (0.03) for the cross-validation study (eFigure 1 in the Supplement) and 0.74 for the testing set (Figure 3).

### Comparison of the Performances of the Conventional Screening Method and the Current Models

Table 2 shows the comparison of representative statistics obtained from each model: the all-elements model, the 5-elements model, and the NICE guideline’s recommendation for CT scans. The sensitivity and specificity of the NICE guidelines were 72.0% (95% CI, 57.5%-83.8%) and 73.3% (95% CI, 68.7%-77.7%), respectively, and those of the all-elements model were 74.0% (95% CI, 59.7%-85.4%) and 74.9% (95% CI, 70.2%-79.3%), respectively. There were no statistically significant differences between this model and the NICE guidelines using the McNemar test (*P* = .80 and *P* = .55, respectively). The McNemar test was also conducted for the NICE guidelines and the 5-elements model. The sensitivity was 74.0% (95% CI, 59.7%-85.4%), and the specificity was 70.1% (95% CI, 65.1%-74.7%), and there were no statistically significant differences between them (*P* = .81 and *P* = .22, respectively).

**Table 2.** Comparison of Representative Statistics That Were Predicted With the Present Machine Learning Models and the NICE Guidelines on the Testing Set

Model	Sensitivity (95% CI)	Specificity (95% CI)	PPV (95% CI)	NPV (95% CI)	Positive likelihood ratio (95% CI)	Negative likelihood ratio (95% CI)
NICE guidelines	72.0 (57.5-83.8)	73.3 (68.7-77.7)	26.7 (19.4-35.0)	95.1 (91.9-97.3)	2.70 (2.12-3.44)	0.38 (0.24-0.60)
XGBoost						
All-elements model	74.0 (59.7-85.4)	74.9 (70.2-79.3)	28.5 (20.9-37.0)	95.5 (92.5-97.6)	2.95 (2.32-3.76)	0.35 (0.22-0.56)
5-Elements model	74.0 (59.7-85.4)	70.1 (65.1-74.7)	25.0 (18.3-32.8)	95.2 (92.0-97.4)	2.47 (1.97-3.10)	0.37 (0.23-0.60)

Abbreviations: NICE, National Institute for Health and Care Excellence; NPV, negative predictive value; PPV, positive predictive value; XGBoost, extreme gradient boosting.

## Discussion

The purpose of this study was to establish a machine learning–based triage system that can detect tICH in patients with head injury using information that can be collected by the ambulance crews at the scene of trauma to select an optimal medical institution for each patient. Among several machine learning algorithms, XGBoost showed a comparatively better prediction performance. The XGBoost model with all available features was able to predict tICH with a sensitivity of 74.0%, a specificity of 74.9%, and an ROC-AUC of 0.80. In addition, a feature importance analysis revealed that 5 features (eg, disorientation, E-GCS, high-energy head trauma, head trauma scar, and pupil abnormalities) were likely to be associated with an accurate prediction. Another model using only these 5 features showed relatively accurate predictions, with a sensitivity of 74.0%, a specificity of 70.1%, and an ROC-AUC of 0.74.

The current process for a patient with head trauma is as follows: (1) ambulance crews judge the severity of the patient’s trauma and decide where to hospitalize them; (2) after transportation, physicians evaluate the necessity of CT scanning through a physical examination; and (3) the patient undergoes CT scanning and may be transported to another hospital. Although well-established tICH screening tools (such as the NICE guidelines) are useful in step 2, to our knowledge there is no established way to properly triage patients in step 1. As the functional outcomes of patients with head injury worsen when their transportation is delayed,^5^ the transport time in step 3 should be reduced by constructing a reliable field triage tool. In general, the severity of patients’ TBI is classified according to their GCS scores.^35^ However, the GCS score is not reliable because it is easily affected by environmental issues, such as alcohol or drug intake, and it tends to underestimate the severity of patients’ TBI because intracranial mass lesions do not immediately affect the patient’s intracranial status, especially among older patients.^36^ Ter Avest et al^37^ tried to detect elevated intracranial pressures with a combination of the GCS score, pupil reaction, and the Cushing triad (bradycardia, irregular breathing pattern, and hypertension), but the sensitivity and ROC-AUC were relatively low (36.8% and 0.65, respectively). We added the crush severity, head trauma scars, and the neurologic findings, such as paresis and seizure (which were easily collectable by ambulance crews at the site), to these features to obtain a new model. These additional features, combined with machine learning techniques, allowed us to achieve a more accurate prediction model compared with previous models.

One of the major advantages of applying artificial intelligence to clinical practice is that it can substitute partially, if not completely, for the assessment by physicians.^38^ During our investigation, we used the NICE guidelines’ CT scan recommendation criteria as a representation of physicians’ assessment and compared its screening performance with those of our present models. As a result, the XGBoost models achieved a predictive accuracy as good as that of the NICE guidelines (Table 2). Seemingly, the sensitivities calculated in our testing set were worse than those of conventional tools, such as the Canadian CT Head Rule (CCHR)^6^ and New Orleans Criteria (NOC),^7^ which were reported to be nearly 100%. Several possible reasons could exist for this finding. First, the CCHR and NOC excluded patients with mild head trauma without loss of consciousness, amnesia, or confusion, which would have resulted in increasing their sensitivity. Second, the outcome was set to be clinically significant brain injuries, which did not include minor tICHs. In a report by Svensson et al,^39^ in which the screening outcome was set as all tICHs, the sensitivity and specificity were, respectively, 87.1% and 35.7% with the CCHR, 97.1% and 3.4% with the NOC, and 75.7% and 58.0% with the NICE guidelines. Considering the trade-off of the sensitivity and specificity on the ROC curve (Figure 3), the performance of our machine learning model is assumed to be as good as these screening tools. The evaluation results of the testing cohort shown in Table 2 also support this assumption. In our model, the PPV, which is another important statistic for imbalanced data, was also comparable to that of the NICE guidelines. Although the improvement in field triage accuracy has been desired in emergency medicine,^40,41,42^ our present prediction model will possibly allow emergency crews, who are not as specialized as physicians, to triage as well as physicians, even with incomplete data. Furthermore, whereas conventional tools output only binary outcomes and yield a single sensitivity, specificity, and PPV value, our model can yield different sensitivity, specificity, and PPV values by changing the cutoff value to adapt to the situation needed, which is another strength of our model.

Regarding the analysis method, while previous screening tools have been established using logistic regression, which is based on linear separation, decision trees can set more than 1 boundary at each variable and can make it possible to classify objects with more complexity. Although complex classification algorithms tend to overfit only training data and will not perform well on test data, several ensemble learning methods, such as random forest and gradient boosting, have been shown to improve their predictive accuracy by preventing overfitting.^43,44^ In our study, XGBoost, as a state-of-the-art tree-based gradient boosting algorithm, allowed us to achieve an acceptable prediction performance. Our current models are available on the GitHub website.^45^ In the future, after proper validation study with a prospective cohort, our models can be converted to web or smartphone applications for ambulance crews to calculate patients’ risk.

### Limitations

Our study has some limitations. First, because this was a single-center study and included only patients who were hospitalized and underwent head CT, our data set may not represent the general population of patients with head trauma. In addition, we suggest that our model may be underestimating patients at high risk, based on the calibration plot. To apply our model to clinical practice, we should verify the predictive accuracy using a prospective external validation set and investigate the optimal cutoff value.

Second, the GCS was not used to evaluate the patient’s state of consciousness because it is not routinely recorded in the Japanese emergency medical system. Instead, the Japan Coma Scale^46^ is widely used to obtain the patient’s state of consciousness. Because the Japan Coma Scale can be translated to the combination of E-GCS and disorientation, our model adopted these 2 items as features. These variables might be less accurate than the GCS itself, and the prediction result might have been degraded.

## Conclusions

Machine learning algorithms have achieved good performance as conventional screening tools in detecting tICH. Although conventional screening tools require examination by a physician, our proposed models require only pretransportation patient information, which can be easily obtained. The results suggest that our proposed prediction models may be useful for constructing a triage system that can be used to assess the optimal institution to which a patient with a head injury should be transported. Further validation with prospective and multicenter data sets is needed.

## Notes

Supplement. **eTable.** The Values of Hyperparameters for Each Algorithm **eFigure 1.** Receiver Operating Characteristic Curves and Precision Recall Curves for the All-Elements XGBoost Model and the 5-Elements XGBoost Model in the Cross Validation **eFigure 2.** Precision Recall Curves for the All-Elements XGBoost Model and the 5-Elements XGBoost Model on the Testing Set **eFigure 3.** The Result of the Feature Importance Analysis
eAppendix.
