# Supplementary material for: A Prehospital Triage System to Detect Traumatic Intracranial Hemorrhage Using Machine Learning Algorithms
Source: JAMA Netw Open. 2022 Jun 10;5(6):e2216393. doi: 10.1001/jamanetworkopen.2022.16393 (PMC9187955; doi:10.1001/jamanetworkopen.2022.16393)
Supplement: Supplement. — eTable. The Values of Hyperparameters for Each Algorithm eFigure 1. Receiver Operating Characteristic Curves and Precision Recall Curves for the All-Elements XGBoost Model and the 5-Elements XGBoost Model in the Cross Validation eFigure 2. Precision Recall Curves for the All-Elements XGBoost Model and the 5-Elements XGBoost Model on the Testing Set eFigure 3. The Result of the Feature Importance Analysis eAppendix. [file jamanetwopen-e2216393-s001.pdf]

Supplementary Online Content

Abe D, Inaji M, Hase T, et al. A prehospital triage system to detect traumatic intracranial hemorrhage using machine learning algorithms. *JAMA Netw Open.* 2022;5(6):e2216393. doi:10.1001/jamanetworkopen.2022.16393

eTable. The Values of Hyperparameters for Each Algorithm
eFigure 1. Receiver Operating Characteristic Curves and Precision Recall Curves for the All-Elements XGBoost Model and the 5-Elements XGBoost Model in the Cross Validation
eFigure 2. Precision Recall Curves for the All-Elements XGBoost Model and the 5-Elements XGBoost Model on the Testing Set
eFigure 3. The Result of the Feature Importance Analysis
eAppendix.

This supplementary material has been provided by the authors to give readers additional information about their work.

© 2022 Abe D et al. *JAMA Network Open.*

eTable. The Values of Hyperparameters for Each Algorithm

Algorithm / hyperparameter	the values of hyperparameters
XGBoost	
learning_rate	(0.0001, 0.001, 0.01, 0.1, 0.2, 0.3, 1)
max_depth	(1, 2, 4, 6, 8, 10, 12)
n_estimators	(100, 300, 1000)
gamma	(0, 0.2, 0.4, 0.6, 0.8, 1)
reg_lambda	(0.1, 0.5, 1)
reg_alpha	(0, 0.1, 0.5)
subsample	(0.2, 0.3, 0.5, 0.7, 0.9, 1.0)
colsample_bytree	(0.2, 0.3, 0.4, 0.6, 0.8, 1.0)
min_child_weight	(2, 4, 6, 8)
Random Forest	
criterion	(“gini”, “entropy”)
n_estimators	(40, 60, 80, 100, 200)
max_features	((1, 3, 5, 7, 9), “auto”, “log2”)
max_depth	(1, 3, 5, 7, 9, 11, 13, “None”)
min_samples_leaf	(1, 3, 5, 7, 9)
SVM	
kernel	(“linear”, “rbf”)
C	(0.01, 0.1, 1, 10, 100)
gamma	(0.01, 0.1, 1, 10, 100)
Logistic regression	
C	(0.001, 0.005, 0.01, 0.02, 0.03, 0.05, 0.1, 0.2, 0.3, 0.5, 1, 5, 10, 100)
max_iter	(50, 100, 300, 500, 1000)
penalty	(“l1”, “l2”, “elasticnet”, “none”)
solver	(“newton-cg”, “lbfgs”, “liblinear”, “sag”, “saga”)
l1_ratio	(0.2, 0.4, 0.6, 0.8)

© 2022 Abe D et al. *JAMA Network Open.*

eFigure 1. Receiver Operating Characteristic Curves and Precision Recall Curves for the All-Elements XGBoost Model and the 5-Elements XGBoost Model in the Cross Validation

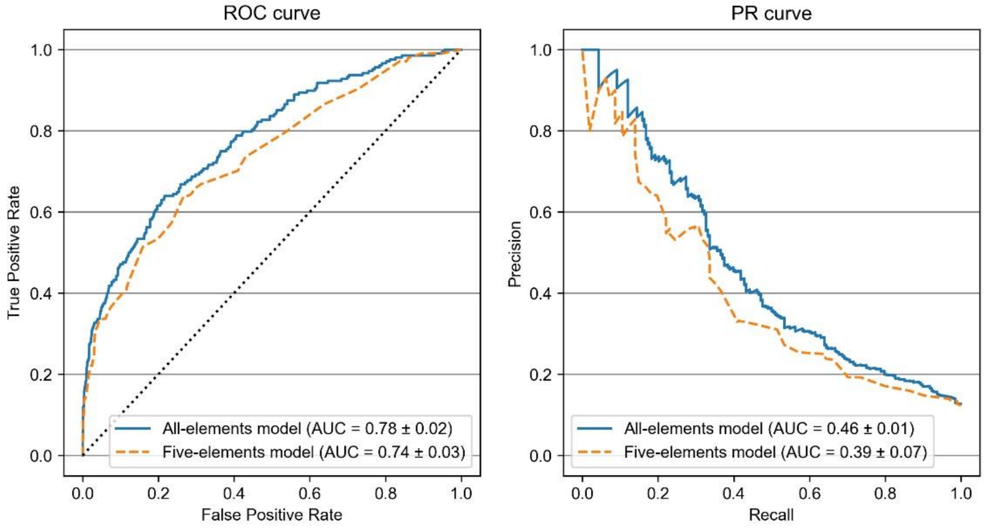

© 2022 Abe D et al. *JAMA Network Open.*

eFigure 2. Precision Recall Curves for the All-Elements XGBoost Model and the 5-Elements XGBoost Model on the Testing Set

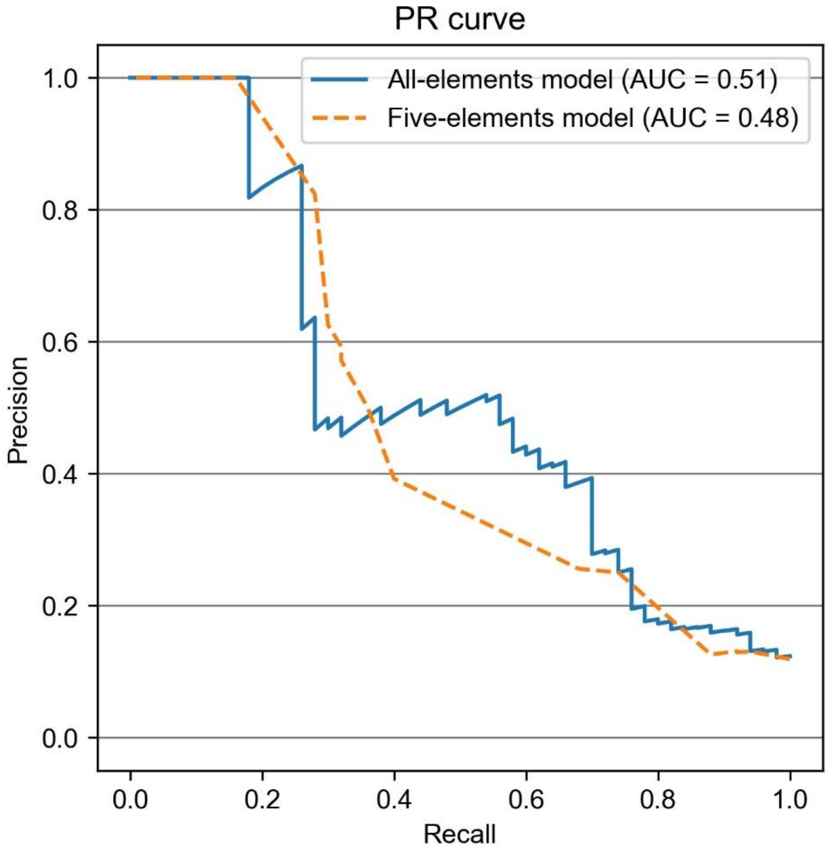

© 2022 Abe D et al. *JAMA Network Open.*

eFigure 3. The Result of the Feature Importance Analysis

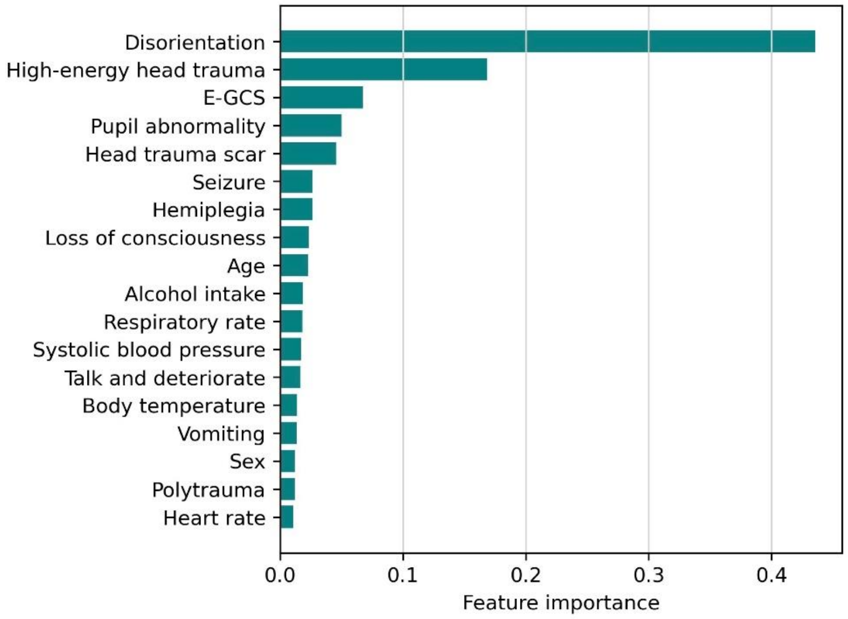

© 2022 Abe D et al. *JAMA Network Open.*

eAppendix

The Glasgow Coma Scale

The Glasgow Coma Scale is used to assess the depth of coma and consciousness. It is calculated by summing points in the following three categories.

Eye opening response

- Spontaneous: 4 points
- To sound: 3 points
- To pain: 2 points
- Never: 1 point

Best Verbal Response

- Oriented: 5 points
- Confused conversation: 4 points
- Inappropriate words: 3 points
- Incomprehensible sounds: 2 points
- None: 1 point

Best Motor Response

- Obeys commands: 6 points
- Localized pain: 5 points
- Flexion – withdrawal: 4 points
- Flexion – abnormal: 3 points
- Extension: 2 points
- None: 1 point

NICE guidelines' criteria for performing a head CT scan

1)For adults who have sustained a head injury and have any of the following risk factors, perform a CT head scan within 1 hour of the risk factor being identified:

- GCS less than 13 on initial assessment in the emergency department.
- GCS less than 15 at 2 hours after the injury on assessment in the emergency department.
- Suspected open or depressed skull fracture.
- Any sign of basal skull fracture (haemotympanum, 'panda' eyes, cerebrospinal fluid leakage from the ear or nose, Battle's sign).
- Post-traumatic seizure.
- Focal neurological deficit.

© 2022 Abe D et al. *JAMA Network Open.*

- More than 1 episode of vomiting.

2)For patients (adults and children) who have sustained a head injury with no other indications for a CT head scan and who are having anticoagulant treatment, perform a CT head scan within 8 hours of the injury.

The definition of high-energy head trauma in NICE guidelines

Pedestrian struck by motor vehicle, occupant ejected from motor vehicle, fall from a height of greater than 1 meter of more than 5 stairs, diving accident, high-speed motor vehicle collision, rollover motor accident, accident involving motorized recreational vehicles, bicycle collision, or any other potentially high-energy mechanism

© 2022 Abe D et al. *JAMA Network Open.*
